# Confirming TDP2 mutation in spinocerebellar ataxia autosomal recessive 23 (SCAR23)

**DOI:** 10.1212/NXG.0000000000000262

**Published:** 2018-08-01

**Authors:** Guido Zagnoli-Vieira, Francesco Bruni, Kyle Thompson, Langping He, Sarah Walker, Arjan P.M. de Brouwer, Robert Taylor, Dmitriy Niyazov, Keith W. Caldecott

**Affiliations:** From the Genome Damage and Stability Centre (G.Z-V., K.W.C.), University of Sussex, Falmer, Brighton, United Kingdom; Wellcome Centre for Mitochondrial Research (F.B., K.T., L.H., R.T.), Institute of Neuroscience, Newcastle University, Tyne, United Kingdom; Sussex Drug Discovery Centre (S.W.), University of Sussex, Falmer, Brighton, United Kingdom; Department of Human Genetics (A.P.M.d.B.), Donders Institute for Brain, Cognition and Behaviour, Radboud University Medical Center, Nijmegen, The Netherlands; and Medical Genetics (A.P.M.d.B., D.N.), Ochsner Health Center for Children, New Orleans, LA.

## Abstract

**Objective:**

To address the relationship between mutations in the DNA strand break repair protein tyrosyl DNA phosphodiesterase 2 (TDP2) and spinocerebellar ataxia autosomal recessive 23 (SCAR23) and to characterize the cellular phenotype of primary fibroblasts from this disease.

**Methods:**

We have used exome sequencing, Sanger sequencing, gene editing and cell biology, biochemistry, and subcellular mitochondrial analyses for this study.

**Results:**

We have identified a patient in the United States with SCAR23 harboring the same homozygous *TDP2* mutation as previously reported in 3 Irish siblings (c.425+1G>A). The current and Irish patients share the same disease haplotype, but the current patient lacks a homozygous variant present in the Irish siblings in the closely linked gene *ZNF193,* eliminating this as a contributor to the disease. The current patient also displays symptoms consistent with mitochondrial dysfunction, although levels of mitochondrial function in patient primary skin fibroblasts are normal. However, we demonstrate an inability in patient primary fibroblasts to rapidly repair topoisomerase-induced DNA double-strand breaks (DSBs) in the nucleus and profound hypersensitivity to this type of DNA damage.

**Conclusions:**

These data confirm the *TDP2* mutation as causative for SCAR23 and highlight the link between defects in nuclear DNA DSB repair, developmental delay, epilepsy, and ataxia.

DNA is under constant threat from attack by endogenous and exogenous electrophilic molecules,^1^ and DNA topoisomerase enzymes can introduce DNA breaks as abortive intermediates of their activity.^2–4^ Topoisomerase “poisons” such as etoposide inhibit the ligation activity of topoisomerase 2 (TOP2), thereby promoting the formation of abortive DNA double-strand break (DSB) intermediates that require DSB repair. DSBs are repaired in cells by either homologous recombination–mediated repair or by nonhomologous end joining (NHEJ).^2^ The repair of TOP2-induced DSBs by NHEJ involves the enzyme tyrosyl DNA phosphodiesterase 2 (TDP2), which removes trapped topoisomerase peptide from the 5′-termini at the DSB and thereby allows the DNA ends to be ligated.^3–5^ The loss of TDP2 in mouse results in reduced expression of >100 genes in the brain,^6^ and *TDP2* mutation in humans has been associated with intellectual disability, seizures, and ataxia,^6^ a disease now denoted as spinocerebellar ataxia, autosomal recessive 23 (SCAR23). To date, our understanding of SCAR23 has been limited by the availability of only 3 Irish siblings with a mutation in TDP2 and by the lack of availability of fibroblast cell lines from these patients for molecular and cellular characterization. Here, we have addressed these limitations and identified an SCAR23 patient in the United States with the same homozygous *TDP2* mutation as present in the Irish siblings, confirming the association of this disease with mutated *TDP2*. In addition, we have characterized at the molecular and cellular level primary patient fibroblasts from the current SCAR23 patient.

## Methods

### Patient case report

The patient is currently a 6-year-old caucasian boy from the United States presenting with developmental delay, microcephaly, and failure to thrive. His mother and father are aged 29 years and 32 years, respectively, and there is no previous family history of related disease, but there is possible consanguinity (3rd cousins). The patient started walking at the age of 14 months and talking at 3 years, his hearing was reportedly normal, but he did not have auditory brainstem response. The patient is easily fatigued, and his parents reported that he eats excessively, becomes irritable, and falls asleep sometimes for days or up to 2 weeks. He is now below the fifth percentile for weight and 5% for height, with a body mass index at <5%. He has a G-tube and is followed by a dietitian and receives nutritional liquid supplements (PediaSure; Abbott, Lake Forest, IL). He has a history of constipation and is followed up by a gastroenterologist. He has a 12-year-old brother who is also easily fatigued and a 14-year-old maternal half-sister with attention deficit hyperactivity disorder (ADHD).

The patient has difficulty keeping balance and has an ataxic gait. He has been tested several times for abnormalities in blood, urine, and CSF, and by MRI and EEG, and only the latter is abnormal. Seizures began at the age of 5 months, and his EEGs show increased slowing and occasional spikes in the right posterior quadrant, and during drowsiness, he had 2 generalized bursts of polyspike and slow-wave activity at a frequency of 4–5 Hz. The patient's 180K oligoarray, very long chain fatty acids, carbohydrate-deficient transferrin, CSF lactate, and neurotransmitters and plasma amino acids were normal.

The patient exhibited several phenotypes consistent with mitochondrial dysfunction such as hypotonia, low energy, fatigability, hypersomnia, failure to thrive, short stature, constipation, neutropenia, hyponatremia, cardiac arrhythmia, and gastrointestinal dysmotility. Consistent with this, electron transport chain (ETC) studies on muscle biopsy at age 1 year showed a severe reduction of complex I + III and II + III activity, which satisfied the major Walker criteria after correction for increased citrate synthase activity. CoQ10 deficiency was suggested based on the complex I + III and II + III deficiency, and his metabolic tests were abnormal with the high lactate and high lactate/pyruvate ratio. The patient was placed on ubiquinol, carnitine, and leucovorin, and anecdotally, he responded well (particularly to liposomal ubiquinol, the active or reduced form of CoQ10) in terms of energy and developmental progress. He is reportedly no longer sleepy and lethargic and has a stable gait, but is “still behind” in language and cognitive skills and is in special education. He still has idiopathic fevers but has not been admitted to the hospital for 15 months. He does not have as many infections but still has gastric dysmotility. His seizures have reduced in frequency.

#### Standard protocol approvals, registrations, and patient consents

We confirm that we have received approval from an institutional ethics standards committee for this work, and we have written informed consent for research from the guardian of the patient for participation in this study.

#### Exome sequencing and haplotype analysis

Whole-exome sequencing (WES) (trio study) of the patient was performed by GeneDx using the Agilent Clinical Research Exome kit to target the exonic regions and flanking splice junctions of the genome. These targeted regions were sequenced simultaneously by massively parallel (NextGen, Irvine, CA) sequencing on an Illumina HiSeq sequencing system with 100bp paired-end reads. Bidirectional sequence was assembled, aligned to reference gene sequences based on human genome build GRCh37/UCSC hg19, and analyzed for sequence variants in the selected genes or regions of interest using a custom-developed analysis tool (Xome Analyzer). Capillary sequencing was used to confirm all potentially pathogenic variants identified in this individual. Sequence alterations were reported according to the nomenclature guidelines of the Human Genome Variation Society. The WES identified a homozygous splice site mutation (c.425+1G>A) in the *TDP2* gene. For comparison of the current patient with the Irish pedigree previously reported,^6^ we conducted haplotype analysis. Variants were considered for homozygosity if they were (1) covered by at least 4 reads or more, (2) present in 80% of all reads or more, (3) designated as a substitution, (4) uniquely positioned in the human genome, and (5) present in the exome data of both individuals. Homozygous regions were determined using a sliding window, accepting 2 or less homozygous variants per 10 variants assessed.

#### Mitochondrial preparation and subcellular fractionation

Mitochondria were prepared as described previously,^7^ with few modifications. HeLa cells and fibroblasts (control and patient) were harvested, resuspended in homogenization buffer (HB [0.6 M mannitol, 10 mM Tris-HCl pH 7.4, 1 mM (ethylene glycol-bis(β-aminoethyl ether)-N,N,N′,N′-tetraacetic acid) (EGTA), 0.1% bovine serum albumin (BSA) (wt/vol]), and subjected to differential centrifugation. Mitochondria were pelleted at 11.000*g* for 10 minutes at 4°C and resuspended in HB; the postmitochondrial supernatant was retained after centrifugation (“post-mito spin”). For submitochondrial fraction preparation, HeLa cell mitochondria (300 µg) were treated with 1.6 µg of proteinase K on ice for 30 minutes, followed by the addition of 5 mM phenylmethanesulfonyl fluoride (PMSF). This fraction was pelleted at 11.000*g* for 10 minutes at 4°C and resuspended in HB. Mitoplasts were obtained by resuspending PK-treated mitochondria in 9 volumes of 10 mM Tris-HCl (pH 7.4) and treated with PK, as described earlier. Inner mitochondrial membrane proteins were extracted in the presence of 100 mM Na_2_CO_3_, followed by centrifugation at 100.000*g* for 15 minutes at 4°C. Proteins (30 µg) from each fraction were loaded onto 12% SDS-PAGE gel, transferred to the polyvinylidene difluoride (PVDF) membrane, and analyzed by immunoblotting using primary antibodies to apoptosis inducing factor (AIF) (NEB), eIF4E (Cell Signalling), EF-Tu (custom made), NDUFB8 (Mitosciences), and TDP2 (see Western Blotting, below).

#### Cell culture and vectors

Human A549 cells were grown in Dulbecco Modified Eagle Medium (Gibco, ThermoFisher, Waltham, MA) containing 10% fetal calf serum (FCS), 2 mM glutamine, penicillin (100 units/mL), and streptomycin (100 μg/mL). Human fibroblasts were grown in Minimum Essential Media (Gibco) containing 15% FCS, 2 mM glutamine, penicillin (100 units/mL), and streptomycin (100 μg/mL). All cells were grown at 5% CO_2_ at 37°C. *TDP2*-mutated patient primary human fibroblasts were established from a patient's skin biopsy and were denoted 850-BR. The control human fibroblast cell line 1-BR.^3^ (denoted here for simplicity as 1-BR) was previously derived from an unrelated normal individual and has been described previously.^8^ For complementation experiments, we used 1-BR cells that were immortalized previously with hTERT (denoted as 1-BR hTERT) and a derivative of 850-BR immortalized in the current study by retroviral-mediated hTERT expression and selected in a medium containing 1 µg/ml puromycin (denoted as 850-BR hTERT). For complementation with human *TDP2*, 850-BR hTERT cells were transfected with either empty eGFP-N1 vector or eGFP-N1 construct encoding GFP-tagged human TDP2 (denoted as TDP2-GFP-N1) and stable transfectants selected for 21 days by growth in a medium containing 0.5 mg/mL G418 (Gibco, ThermoFisher, Waltham, MA). TDP2-GFP-N1 was generated by PCR amplification of the human TDP2 ORF using the primers *TDP2*_FW (5′-AAAGAATTCATGGAGTTGGGGAGTTGCCTG-3′) and *TDP2*_RV (5′-AAAGGATCCAATATTATATCTAAGTTGCACAGAAGACC-3′) and subcloning the PCR product into the *Eco*RI/*Bam*HI sites of eGFP-N1.

#### CRISPR/Cas9 gene editing

A549 *TDP2*^−/−^ cells were created as previously reported.^9^ In brief, we used a 17-bp (minus the PAM) RNA sequence targeting TDP2 exon 4 (5′-GTAGAAATATCACATCT-3′), which was selected using the tool E-CRISP (e-crisp.org/E-CRISP/) and cloned into the guide RNA vector #41824 (AddGene). The TDP2 guide construct was cotransfected with hCas9 expressed from plasmid #41815 (AddGene) using Amaxa Nucleofector plataform (Lonza, Basel, Switzerland) Kit T program X-001. Transfected cells were enriched by selection in 1 mg/mL G418 (Thermofisher) for 5 days before isolation of single clones and screening for loss of TDP2 expression by Western blotting.

#### Sanger sequencing

DNA was extracted from 850-BR cells using the DNeasy Blood & Tissue kit (Qiagen, Manchester, UK). PCR reactions used Phusion HF-DNA Polymerase (NEB) and the following primers: *TDP2*_FW GCCAGTGTTGACCTAACCAATGAAGA; *TDP2*_RV CTGTAGAAATATCACATCTGGGCTGTACC; ZSCAN9_FW ATGAAGTAACCAAGACTGAGGACAGAGAG; and ZSCAN9_RV AGACCAGCTCAGCCACTGTGTGGATCT. PCR products were purified before sequencing using a QIAquick PCR purification kit (Qiagen).

#### Western blotting

Anti-TDP2 antibody was used at 1:5000 in Western blotting and has been described previously.^10^ Anti-Actin (Sigma A4700) was used at 1:2000. Western blot assessment of OXPHOS components in patient primary fibroblasts was conducted as described previously,^11^ using antibodies against NDUFB8 (Abcam cat# ab110242), SDHA (Abcam cat# ab14715), UQCRC2 (Abcam cat# ab14745), COXI (Abcam cat# ab14705), ATP5A (Abcam cat# ab14748), SDHB (Abcam cat# ab14714), COX II (Abcam cat# 110258), and VDAC1 (Abcam cat# ab14734).

#### Clonogenic survival assays

1-BR and 850-BR primary fibroblasts cells were plated onto feeder layers (see below) and 3 hours later treated with indicated concentrations of etoposide for 21 days to allow the formation of macroscopic colonies, which were rinsed in phosphate buffered saline (PBS) and fixed/stained in 70% ethanol/1% methylene blue. A549 and *TDP2*^−/−^ A549 were plated 4 hours before treatment with the indicated concentrations of etoposide for 12 days and stained as described earlier. The surviving fraction at each dose was calculated by dividing the average number of colonies (>50 cells) in treated dishes by the average number in untreated dishes. For feeder layers, 1-BR cells were irradiated (35 Gy) and plated 24 hours before use at 5 × 10^4^ cells/10 cm dish.

#### Tyrosyl DNA phosphodiesterase assays

Whole-cell extract (WCE) was prepared by resuspension of 1-BR or 850-BR primary fibroblast cell pellets (1×10^6^cells) in 100µL lysis buffer (40 mM Tris/HCl pH 7.5, 100 mM NaCl, 0.1% Tween-20, 1 mM DTT, 1 mM PMSF, 1x EDTA free protease cocktail inhibitor), followed by 30 minutes of incubation on ice and mild sonication. The WCE was clarified by centrifugation for 10 minutes at 4°C at 16000g in a microfuge and the protein concentration quantified using the bicinchoninic acid (BCA) assay reagent (ThermoFisher). Clarified WCE (15µg total protein) was incubated with 40 nM TDP2 substrate (Cy5-5′Tyrosine-ssDNA_19_-BHQ) or TDP1 substrate (BHQ-ssDNA_13_-3′Tyrosine-Cy5) in reaction buffer (50 mM Tris/HCl pH8.0, 10 mM MgCl_2_, 80 mM KCl, and 1 mM DTT, 0.05% Tween-20) in a total volume of 6µL at room temperature, and Cy5 fluorescence was measured at 640 nm at the indicated time intervals on a BMG PHERAstar plate reader.

### DSB repair assays

Cells were grown on coverslips until confluent and then treated for 30 minutes with 25µM etoposide or irradiated with x-rays (2 Gy). After treatment, cells were rinsed and fixed for 10 minutes in PBS containing 4% paraformaldehyde at the indicated time points. Cells were permeabilized (20 minutes in PBS-0.2% Triton X-100), blocked (1 hour in PBS-5% BSA), and incubated with anti-γH2AX (Millipore, 05-636, 1:2500) and anti-CENP-F (Abcam, ab5, 1:2500) antibodies for 3 hours in PBS containing 5% BSA. Cells were then washed (3 × 5 minutes in PBS containing 0.1%Tween-20), incubated for 1h with the corresponding Alexa Fluor conjugated secondary antibody (1:1000, 5% BSA), and washed again as described earlier. Finally, cells were counterstained with DAPI (Sigma, Gillingham, UK) and mounted in VECTASHIELD (Vector Labs, Peterborough, UK). Images were acquired on an automated wide-field microscopy Olympus ScanR system (motorized IX83 microscope) with ScanR Image Acquisition and Analysis Software, 20×/0.45 (LUCPLFLN 20×PH) dry objectives, and Hamamatsu ORCA-R2 digital CCD camera C10600. For the analysis of G0/G1 cells in patient primary fibroblasts, only CENP-F-negative cells were scored. For A549 and A549 *TDP2*^−/−^, cells were gated to the G1 population according to the DAPI profile of ScanR Image Analysis Software. For patient fibroblast complementation experiments, cells were gated accordingly to eGFP expression on ScanR Analysis Software, in addition to the G1 population according to the DAPI content.

## Results

**Figure 1:**
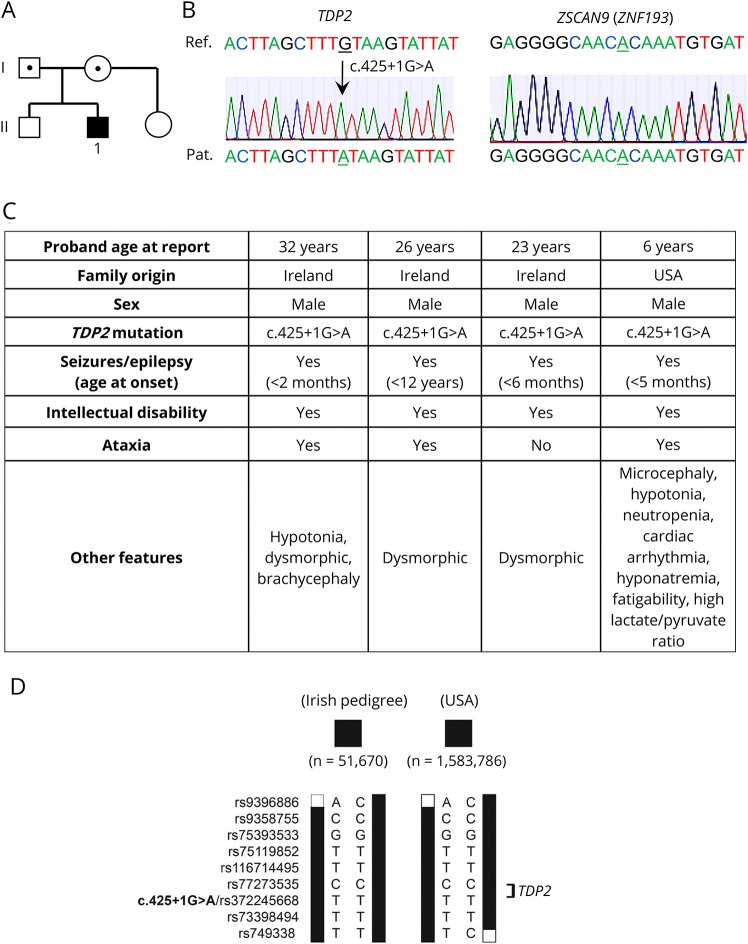
*TDP2* splice site mutation in an individual from the United States with SCAR23 (A) Pedigree analysis. White symbols: wild-type *TDP2*. Black symbols: homozygosity for the *TDP2* splice site mutation c.425+1G>A (IVS3+1G>A) in the proband (“1”). Dotted symbols, heterozygosity for c.425+1G>A (IVS3+1G>A). (B) Sanger sequencing of the proband, demonstrating (left) the homozygous *TDP2* mutation c.425+1G>A (IVS3+1G>A) and (right) the absence of the ZSCAN9 variant c914A>G. Patient sequences are shown at the bottom, and reference sequences are shown at the top. The 2 nucleotides relevant to the mutations are underlined. (C) Pathologic features of the Irish and US patients. (D) Haplotypes of Irish patients (51670; Irish pedigree) and the current patient from the United States (1583786) carrying the homozygous c.425+1G>A variant in *TDP2*. Only the variants bordering and directly within the homozygous regions and with a minor allele frequency of 5% are shown. Variants are indicated by their accession number in the dbSNP database, and the position of c.425+1G>A is in bold. The minimal overlapping region is delimited by rs9396886 and rs749338 and is 15.1 Mb in size. Black bars represent the homozygous haplotype as inferred from exome sequencing data. *TDP2 =* tyrosyl DNA phosphodiesterase 2.

We recently described mutations in *TDP2* in three Irish patients from the same family with intellectual disability, seizures, and ataxia, a disease now denoted as spinocerebellar ataxia 23 (SCAR23)^6^. Here, we describe a 6-year-old patient in the United States with very similar pathology including developmental delay, epilepsy, and ataxia and in whom we identified by whole-exome and Sanger sequencing possesses the same homozygous splice site mutation in *TDP2* (c.425+1G>A) (figure 1, A–C). Whether there is a connection between the current patient and the Irish family is not clear, but a comparison of the WES data from the two families revealed that the two apparently unrelated affected individuals of whom their exome was sequenced share the same homozygous haplotype of ∼15.1 Mb (figure 1D). It is important to note that in contrast to the Irish patients, however, the current patient lacks the mutation c.914A>G (p.His305Arg) in *ZNF193*, a zinc finger protein of unknown function (figure 1B, right). We previously concluded that this variant was not disease causing in the Irish patients,^6^ and the absence of this variant in the current patient confirms that this and also that *TDP2* mutation is the cause of SCAR23.

**Figure 2:**
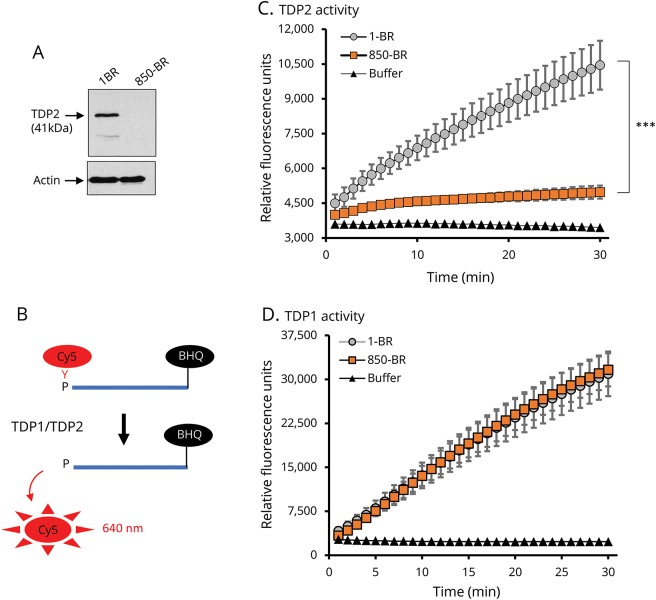
Greatly reduced *TDP2* protein and activity in 850-BR patient fibroblasts (A) TDP2 and actin protein levels in WCE (20 μg total protein) from 1-BR normal and 850-BR patient primary fibroblasts, as measured by immunoblotting. (B) TDP1 and TDP2 biochemical assay. Hydrolysis of a 3′-phosphotyrosyl (TDP1 assay) or 5′-phosphotyrosyl (TDP2 assay) bond releases the associated Cy5 fluorophore and alleviates quenching by the BHQ located on the opposite oligonucleotide terminus, resulting in elevated fluorescence at 640 nm. (C) Real-time (0–30 minutes) measurements of 5′-tyrosyl DNA phosphodiesterase activity (TDP2 assay) in reaction buffer (as a negative control) or WCE (15 μg protein) from 1-BR normal or 850-BR patient primary fibroblasts, using the single-stranded oligonucleotide (40 nM) with a 5′-phosphotyrosyl-linked Cy5 fluorophore and 3′-BHQ. (D) Real-time measurements (0–30 minutes) of 3′-tyrosyl DNA phosphodiesterase (TDP1 assay) conducted as above but using a 3′-phosphotyrosyl-linked Cy5 fluorophore and 5′-BHQ. Data are mean of 3 independent experiments ±SEM. Statistical significance was determined by two way analysis of variance (ANOVA). ***p < 0.001. BHQ = black hole quencher; *TDP2 =* tyrosyl DNA phosphodiesterase 2; WCE = whole-cell extract.

To enable additional molecular and cellular analyses of SCAR23, we generated primary fibroblasts (denoted 850-BR) from a skin biopsy kindly provided by the current patient. Western blotting failed to detect TDP2 protein in the 850-BR patient fibroblasts (figure 2A), consistent with the lack of detectable TDP2 protein previously reported in lymphoblastoid cells from the Irish patients.^6^ The absence of detectable TDP2 protein in the lymphoblastoid cell lines was explained by the impact of the *TDP2* mutation on splicing, which resulted in nonsense-mediated decay and greatly reduced (<20%) levels of *TDP2* mRNA.^6^ To confirm the impact of the splice site mutation on TDP2 activity, we used a highly sensitive tyrosyl DNA phosphodiesterase biochemical assay. This assay uses a single-stranded oligonucleotide substrate in which hydrolytic release of a Cy5-labeled tyrosine moiety present on one terminus of the oligonucleotide results in increased fluorescence due to evasion of a black hole quencher present on the opposite terminus (figure 2B). By situating the fluorescent-labeled tyrosine on either the 3′ or 5′ terminus, this assay can detect TDP1 or TDP2 activity, respectively. Although whole-cell extract from normal 1-BR human fibroblasts exhibited robust 5′-tyrosyl DNA phosphodiesterase activity, 850-BR patient fibroblasts did not (figure 2C). The small amount of activity observed in 850-BR cell extract, compared with reactions supplemented with reaction buffer alone, reflects nucleases in the cell extract rather than residual TDP2 activity because similar results were observed when we used cell extract from human cells in which TDP2 was deleted by CRISPR/Cas9 (unpublished observations). The lack of TDP2 activity in patient cells did not reflect differences in the technical quality of cell extracts because the level of TDP1 activity in these extracts was similar to that in normal human 1-BR fibroblasts (figure 2D). We conclude from these data that the *TDP2* splice site mutation greatly reduces and most likely ablates both TDP2 protein and activity in 850-BR patient fibroblasts.

**Figure 3:**
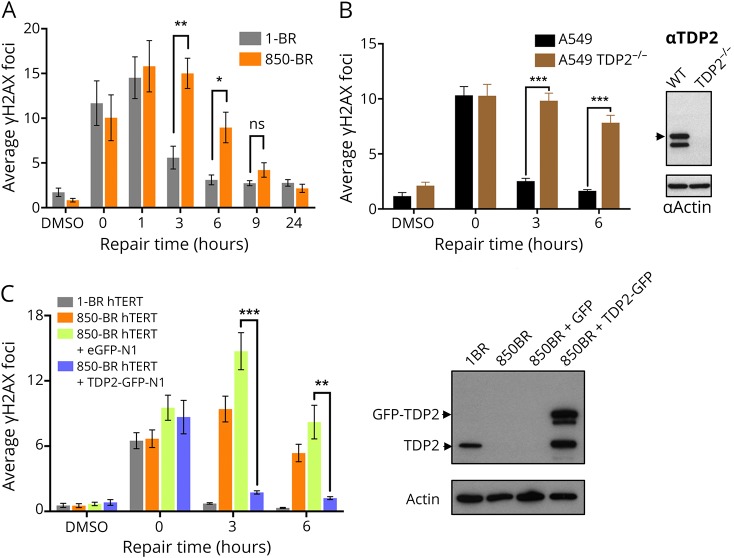
Reduced DSB repair in TDP2-mutated patient fibroblasts and A549 cells after topoisomerase 2-induced DNA damage (A) DSBs were measured by γH2AX immunostaining in normal 1-BR and patient 850-BR primary fibroblasts before and after treatment for 30 minutes with DMSO vehicle or 25 μM etoposide (“0”), followed by subsequent incubation in a drug-free medium for the indicated repair periods. (B) DSBs were measured as above, in wild-type A549 cells and in A549 cells in which *TDP2* was deleted by CRISPR/Cas9 gene editing (*TDP2*^−/−^ A549). The level of TDP2 and actin (loading control) in the cell lines used for these experiments is shown by Western blotting on the right. (C) DSBs were measured as above in hTERT-immortalized 1-BR fibroblasts and 850-BR fibroblasts and in the latter cells after transfection with the empty GFP vector or vector encoding recombinant human TDP2-GFP. The level of TDP2 and actin (loading control) in the cell lines used for these experiments is shown by Western blotting on the right. Data are mean ± SEM of 3 independent experiments, and statistically significant differences were determined by the *t* test (ns = not significant, *p < 0.05; **p < 0.01; ***p < 0.001). DSB = double-strand break; GFP = green fluorescent protein; TDP2 = tyrosyl DNA phosphodiesterase 2.

**Figure 4:**
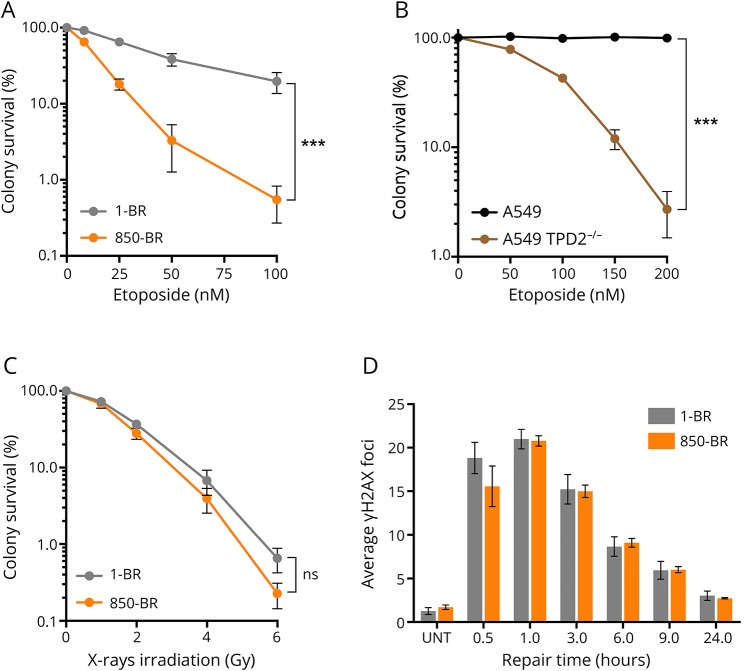
Hypersensitivity of TDP2-mutated patient fibroblasts and A549 cells to DNA damage induced by etoposide but not γ-rays (A) Clonogenic survival of 1-BR normal and 850-BR patient primary fibroblasts in a medium containing the indicated concentrations of etoposide. (B) Clonogenic survival of wild-type A549 and *TDP2*^−/−^ A549 cells in a medium containing the indicated concentrations of etoposide. (C) Clonogenic survival of 1-BR normal and 850-BR patient primary fibroblasts as above, following γ-irradiation. (D), DSBs were measured by γH2AX immunostaining in normal 1-BR and patient 850-BR primary fibroblasts before and after the indicated periods after γ-irradiation (2 Gy). Data are mean ± SEM of 3 independent experiments, and statistically significant differences were determined by two-way ANOVA (ns = not significant, ***p < 0.001). TDP2 = tyrosyl DNA phosphodiesterase 2.

Next, we addressed the impact of the TDP2 splice site mutation on nuclear DSB repair rates in 850-BR patient fibroblasts using immunofluorescent detection of γH2AX as an indirect measure of DSBs.^12^ Although similar levels of nuclear γH2AX foci were present in normal and patient fibroblasts immediately after treatment with etoposide for 30 minutes, the levels of these γH2AX foci decreased far more slowly in the patient fibroblasts during subsequent incubation in a drug-free medium (figure 3A). However, DSB repair was completed in patient cells within 8–24 hours, consistent with the established existence of alternative, nuclease-dependent mechanisms for repair of TOP2-induced DSBs in human cells.^4,13,14^ Similar results were observed in human A549 cells in which *TDP2* was mutated by CRISPR/Cas9 gene editing, confirming the importance of TDP2 for repair of TOP2-induced DSBs (figure 3B). More importantly, complementation of 850-BR cells with expression construct encoding recombinant human TDP2 restored normal rates of nuclear DSB repair, confirming that the DNA repair defect in the patient fibroblasts was the result of the *TDP2* mutation (figure 3C). This defect in DSB repair was accompanied by cellular hypersensitivity to TOP2-induced DSBs because both 850-BR patient fibroblasts and *TDP2*^−/−^ A549 cells were hypersensitive to etoposide in clonogenic survival assays (figure 4, A and B). In contrast to etoposide-induced DSBs, both DSB repair rates and levels of cell survival were normal in 850-BR patient fibroblasts after treatment with ionizing radiation (figure 4, C and D), confirming the specificity of the DNA repair defect in the patient's cells for DNA breaks induced by TOP2.

**Figure 5:**
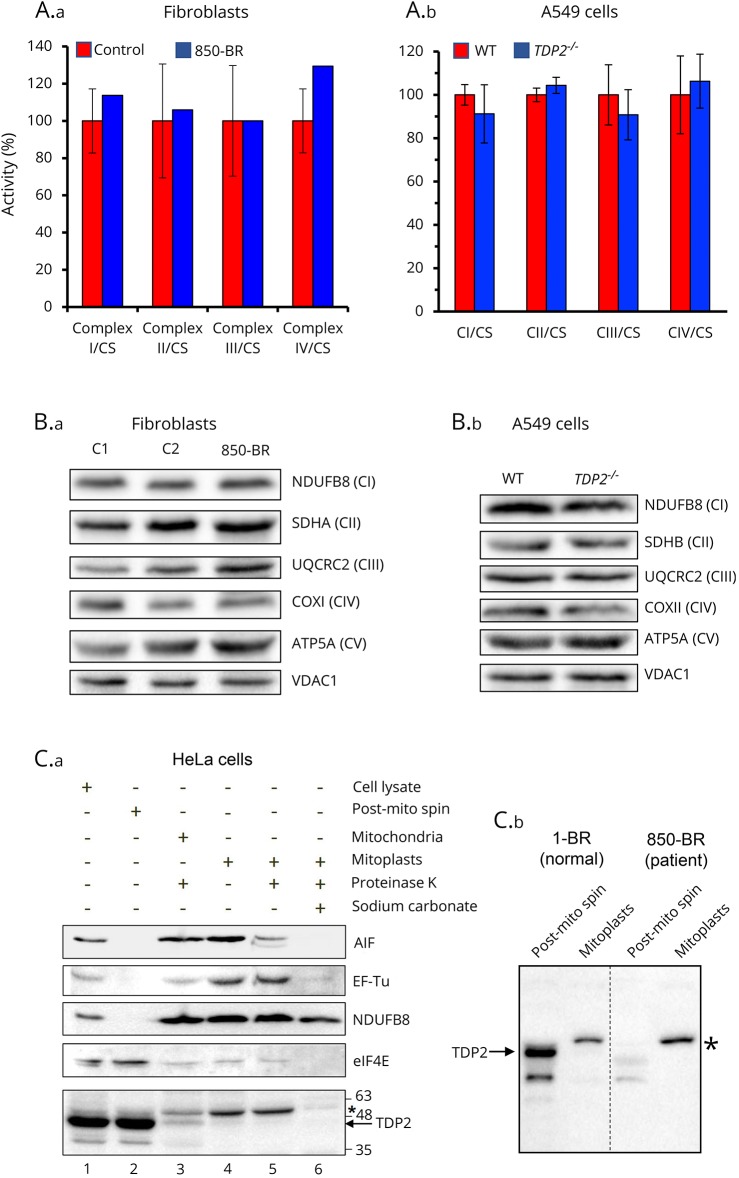
Mitochondrial functionality in normal and TDP2-mutated cells (A) Activity of mitochondrial respiratory complexes in normal (red bars) and patient 850-BR (blue bars) primary human fibroblasts (A.a) and in wild-type human A549 (red bars) and TDP2^−/−^ A549 cells (blue bars) (A.b) was determined as previously described.^15^ The mean enzyme activities in control cells (n = 8) are set to 100%, and error bars represent SD. (B) Levels of mitochondrial respiratory complex proteins in age-matched control fibroblasts (C1 and C2) and patient 850-BR fibroblasts (B.a) and in wild-type A549 cells and TDP2^−/−^ A549 cells (B.b), as measured by immunoblotting for subunits of CI (NDUFB8), CII (SDHA), CIII (UQCRC2), CIV (COXI), and CV (ATP5A) and using VDAC1 as a mitochondrial loading control. (C) Subcellular localization of TDP2 in HeLa cells (C.a) and in normal (1-BR) and patient (850-BR) primary human fibroblasts (C.b). Left, cell-equivalent amounts of HeLa total cell lysate (30 g total protein; lane 1), cell lysate depleted of mitochondria (“post-mito spin”; lane 2), mitochondria treated with proteinase K (to remove proteins associated with the outer membrane; lane 3), mitoplasts (mitochondrial matrix plus inner membrane proteins; lane 4), mitoplasts treated with proteinase K (lane 5), and inner membrane mitochondrial proteins (extracted with sodium carbonate; lane 6) were immunoblotted for TDP2 and for protein markers of the cytosol (eIF4E) and each of the mitochondrial compartments, intermembrane space (AIF), mitochondrial matrix (EF-Tu), and inner mitochondrial membrane (NDUFB8). Right, cell-equivalent amounts of 1-BR or 850-BR fibroblast total cell lysates (30μg protein) depleted of mitochondria (“post-mito spin”) and of mitoplasts were immunoblotted for TDP2 as above. The position of full-length TDP2 (arrow) and a nonspecific band detected by the antibody (asterisk; *) are indicated. TDP2 = tyrosyl DNA phosphodiesterase 2.

Finally, because analyses of muscle biopsy from the patient (see the case report) suggested the presence of possible defects in the ETC, we examined 850-BR patient fibroblasts for defects in mitochondrial function. However, the patient fibroblasts failed to exhibit significant defects in respiratory chain complexes in biochemical assays compared with a range of different normal (control) human fibroblasts (figure 5A.a). Similar results were observed when we compared wild-type A549 cells with A549 cells in which *TDP2* was mutated by gene editing (figure 5A.b). We also failed to identify any impact of the *TDP2* mutation or deletion in 850-BR fibroblasts and A549 cells, respectively, in the level of respiratory chain proteins as measured by immunoblotting (figure 5B).

We next fractionated HeLa cells and both 1-BR and 850-BR fibroblasts into subcellular components, including intact mitochondria and mitoplasts, to examine whether TDP2 is located within the mitochondria and thus in the correct location to repair mitochondrial DNA (figure 5C). Most of the material detected by the anti-TDP2 antibody and which migrated to the position expected for TDP2 was present, as expected, in the cellular fractions containing cytosolic and nuclear proteins (figure 5C.a, lanes 1 and 2). However, a very small amount of this material was detected in intact mitochondria and mitoplasts (figure 5C.a, lanes 3–5). In 1-BR normal human fibroblasts, most of the material detected by anti-TDP2 antibody was again present in the cytosolic/nuclear fraction, and this material was TDP2 because it was absent from parallel preparations from patient 850-BR fibroblasts (figure 5C.b). However, if TDP2 was present in mitoplasts prepared from 1-BR cells, it was below the level of detection in our experiments (figure 5C.b, arrow). We did note the presence of a slower migrating band that was detected by the TDP2 antibody in both HeLa and 1-BR mitoplasts, but this band was also present in parallel preparations from 850-BR patient fibroblasts, indicating that it was not TDP2 (figure 5C.b, asterisk). Collectively, although we cannot rule out the presence of a small amount of endogenous TDP2 in mitochondria, we cannot detect defects in mitochondrial function in SCAR23 patient fibroblasts. Consequently, we suggest that the neurologic disease pathology in SCAR23 is most likely the result of a defect in nuclear DSB repair.

## Discussion

We recently identified *TDP2* mutations in a recessive hereditary genetic disorder associated with intellectual disability, seizures, and ataxia, now denoted as spinocerebellar ataxia autosomal recessive 23 (SCAR23)^6^. In the three Irish siblings originally described, we also identified the mutation c.914AG (p.His305Arg) in *ZNF193,* a zinc finger protein of unknown function, which we concluded was not disease causing but which we could not exclude as a contributor to the disease.^6^ However, in contrast to the Irish patients, the new patient identified in the United States and reported here harbors the same *TDP2*-associated haplotype but lacks the mutation in *ZNF193*, ruling out a contribution of the latter and confirming the *TDP2* mutation as the cause of SCAR23.

The *TDP2*-mutated patient fibroblasts established here are the first such reported and have provided valuable information concerning the molecular and cellular defect in SCAR23. Consistent with TDP2 defects in other cell types, we observed reduced rates of DSB repair and elevated cellular sensitivity after treatment with the TOP2 poison etoposide. These phenotypes were the result of the TDP2 mutation in the patient fibroblasts because they were phenocopied in *TDP2*^−/−^ A549 cells created by gene editing and were complemented by the reintroduction of the wild-type human *TDP2* transgene. Of interest, the magnitude of these phenotypes is greater in patient fibroblasts and gene-edited A549 cells than what we have seen previously with our previous gene-edited cell lines.^9^ We believe that this is because the cell lines reported here are effectively TDP2 null, whereas our previously generated TDP2 gene-edited human cells retained a minor isoform of TDP2 that is expressed at low levels and is primarily cytoplasmic.

It is notable that TOP2 poisons are used widely in the clinic to treat a variety of cancers, and we point out that the use of these agents in patients with TDP2 mutations should be avoided or at the very least treated with extreme caution. Although TDP2-defective cancer cells will be hypersensitive to this type of chemotherapy, normal cells will also be hypersensitive, as illustrated in the current work by the etoposide sensitivity observed in both *TDP2*^−/−^ A549 lung cancer cells and SCAR23 primary fibroblasts.

The presence of mitochondrial dysfunction in the SCAR23 patient described here is suggested by ETC studies and the high lactate and lactate/pyruvate ratio in muscle (see the Patient Case Study in the Methods section). In addition, the current patient exhibits phenotypes consistent with mitochondrial dysfunction such as hypotonia, low energy, and fatigability. It is unclear whether phenotypes consistent with mitochondrial defects are also present in the Irish patients, although none were originally noted.^6^ Unfortunately, neither fibroblasts nor muscle biopsy are available from these patients to address this possibility. The lack of detectable mitochondrial defect in primary skin fibroblasts from the current patient and in gene-edited A549 cells does not support a major role for TDP2 in the repair of mitochondrial DNA. However, the absence of mitochondrial defects in skin fibroblasts from patients with mitochondrial defects in muscle is not uncommon.^15^ Nevertheless, given the dramatic impact of the TDP2 mutation on nuclear DNA repair, we suggest that any association of SCAR23 with mitochondrial disease is most likely an indirect or secondary dysfunction resulting from a nuclear disorder.^16^

## Glossary

DSB: double-strand break
ETC: electron transport chain
FCS: fetal calf serum
NHEJ: nonhomologous end joining
SCAR23: spinocerebellar ataxia, autosomal recessive 23
TDP2: tyrosyl DNA phosphodiesterase 2
WCE: whole-cell extract
WES: Whole-exome sequencing
